# momapy: a Python library to work with molecular maps

**DOI:** 10.1093/bioinformatics/btag352

**Published:** 2026-06-03

**Authors:** Adrien Rougny, Marek Ostaszewski, Venkata Sagatopam

**Affiliations:** Luxembourg Centre for Systems Biomedicine (LCSB), University of Luxembourg, Esch-sur-Alzette, Luxembourg; Luxembourg Centre for Systems Biomedicine (LCSB), University of Luxembourg, Esch-sur-Alzette, Luxembourg; Luxembourg Centre for Systems Biomedicine (LCSB), University of Luxembourg, Esch-sur-Alzette, Luxembourg

## Abstract

**Motivation:**

Molecular maps are graphical representations of the molecular mechanisms underlying biological systems. They are a valuable tool for curating, exchanging, and understanding biological knowledge, and may serve as a backbone for data analysis and modelling. Molecular maps are supported by a rich software ecosystem. However, there are currently no tools that support advanced programmatic analysis and processing of maps, in particular the extraction of the biological concepts they represent or their comparison.

**Results:**

We introduce *momapy*, a generic Python library to work with molecular maps programmatically. At its core, *momapy* allows users to extract and separate the data model of a map from its graphical representation, and perform a variety of base operations on them, including their manipulation and comparison. *momapy* currently supports the SBGN and CellDesigner formats, two of the main standards to represent molecular maps graphically, and can be easily extended to support additional formats and functionalities.

**Availability:**

*momapy* is implemented in Python (RRID:SCR_008394) under a GPLv3 license. The code can be downloaded freely from https://github.com/adrienrougny/momapy and is archived on Zenodo (https://doi.org/10.5281/zenodo.19088611). Complete documentation and a user manual are available at https://adrienrougny.github.io/momapy.

## 1 Introduction

Molecular maps are graphical representations of the molecular mechanisms underlying biological systems. They are detailed, curated, and exchangeable graphical knowledge bases that help to understand the mechanisms driving phenotypes of interest (e.g., diseases (Ostaszewski *et al*. 2019), induced cellular responses (Oda *et al*. 2005, Calzone *et al*. 2008)). They may also support a variety of analyses, including analysis of omics data and dynamical modeling (Niarakis *et al*. 2024).

Molecular maps are supported by a rich software ecosystem. The available tools allow users to edit (CellDesigner™ (Funahashi *et al*. 2008), SBGN-ED (Czauderna *et al*. 2010), Newt (Balci *et al*. 2021)), visualise (MINERVA (Gawron *et al*. 2016)), render (Newt, MINERVA, SBMLDiagrams (Xu *et al*. 2023)), format and convert (libSBGN (van Iersel *et al*. 2012), MINERVA, cd2sbgnml (Balaur *et al*. 2020)), query (STONPy (Rougny *et al*. 2023)) or model (SBGN2AN (Rougny *et al*. 2016), CasQ (Aghamiri *et al*. 2020)) maps. However, operations required for advanced programmatic analysis and processing are not supported by any of these tools. For example, it is currently not possible to extract the biological concepts represented by a map or to compare two maps automatically.

Here we introduce *momapy*, a generic Python library for working with molecular maps programmatically. At its core, *momapy* separates the *model* of a map from its *layout*: while the model of a map describes what biological concepts it represents, its layout describes how they are represented graphically. This distinction is borrowed from the Systems Biology Markup Language (SBML) (Hucka *et al*. 2003), where a model defines a set of reactions and their associated mathematical equations, but may be augmented with a graphical layout using the layout and render packages (Gauges *et al*. 2015, Bergmann *et al*. 2018). *momapy* generalises this distinction and applies it to the Systems Biology Graphical Notation (SBGN) (Le Novere *et al*. 2009) and CellDesigner™ (Funahashi *et al*. 2008), two of the main standard languages to represent maps graphically.

## 2 A *momapy* map: a model, a layout, and a layout-model mapping

In *momapy*, a *map* is made up of three distinct elements: a *model*; a *layout*; and a *layout-model mapping*. The *model* of a map is a structured collection of model elements that encode the biological concepts represented in the map. *momapy* defines a hierarchical data model for each type of map it supports (SBGN Process Description (PD) (Rougny *et al*. 2019), SBGN Activity Flow (AF) (Mi *et al*. 2015), and CellDesigner™ (Funahashi *et al*. 2008), see section Supported map formats for more details), formed of class and subclass relations encoding the biological concepts based on the map type and their hierarchy in the Systems Biology Ontology (Juty and le Novère 2013). The encoded biological concepts include entity pools, processes and modulations for process description types of maps (SBGN PD, CellDesigner™), or activities and modulations for activity types (SBGN AF). The *layout* of a map is a structured collection of layout elements encoding the glyphs (graphical symbols) of the map. Each layout element encodes a specific shape (e.g., a circle, a rectangle with rounded corners) whose attributes define how it may be rendered (e.g., its position, dimensions, fill color). A layout element may itself contain other layout elements, giving a tree-like structure to the layout, similar to DOM-based documents such as HTML or SVG. Finally, the *layout-model mapping* associates each layout element of the map with the model element it represents.

## 3 *momapy*’s features

**Figure 1:**
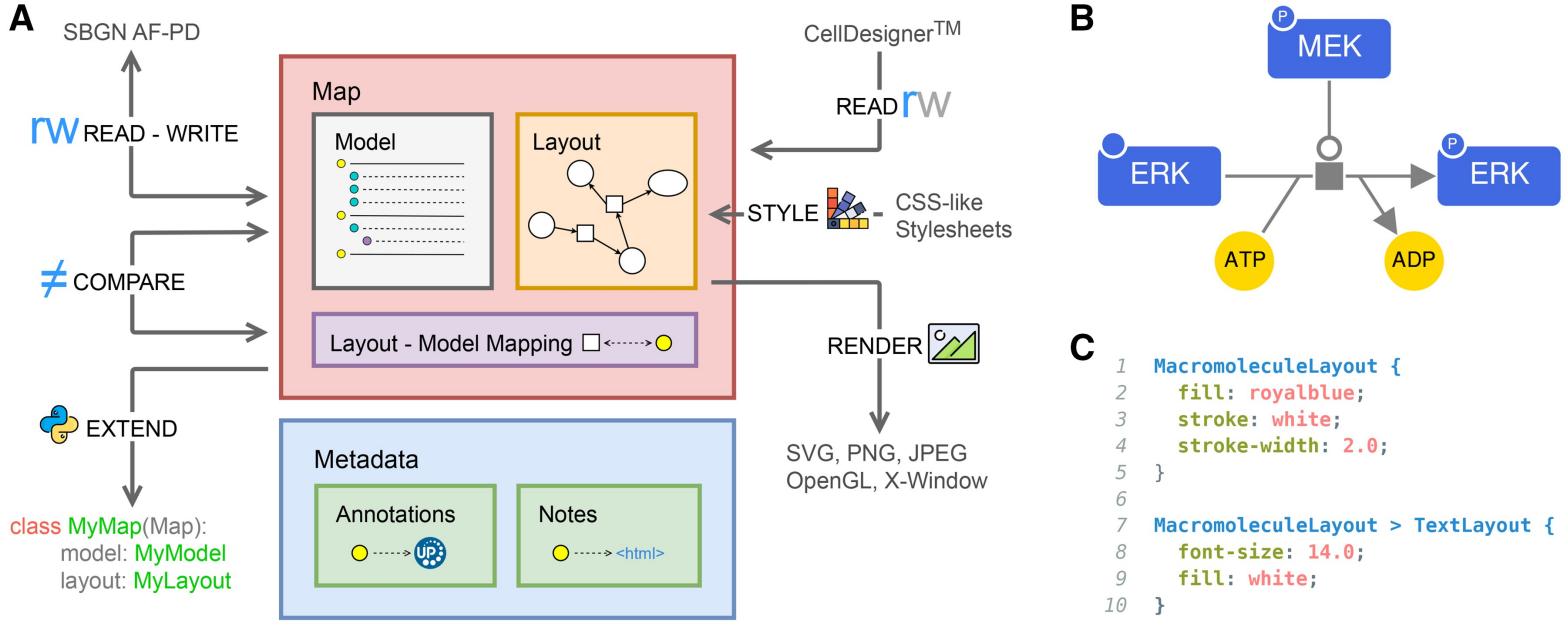
Overview of *momapy*’s features. (A) A graphical summary of *momapy*’s features, showing how a map is formed of a model, a layout, and a layout-model mapping. (B) An example of an SBGN PD map styled and rendered using *momapy*. (C) An excerpt of the CSS-like style sheet used to style the map represented in B.

At its core, *momapy* allows users to extract a map’s model and layout, and work with them programmatically through a variety of features. These are summarised in Fig. 1A, and detailed below.

## 4 Supported map formats

*momapy* currently supports SBGN (PD and AF) and CellDesigner™, two of the main languages used to draw molecular maps. SBGN maps may be read and written from/to SBGN-ML, and CellDesigner™ maps read from the CellDesigner™ exchange format (based on SBML).

## 5 Exploring and manipulating maps

*momapy* elements (maps, models, layouts, and their sub-elements) are made readily available as Python objects, that can be easily explored and manipulated programmatically. Notes and annotations (as defined by SBML) are made accessible outside of the map they describe, complying with the FAIR principles on the separation of metadata from data (Balaur *et al*. 2025).

## 6 Comparing maps

*momapy* elements are implemented as frozen dataclasses so that they can be easily compared programmatically. This implementation also allows users to check whether a *momapy* element belongs to a given set of objects efficiently, which is crucial for some applications where one needs to compare large sets of maps (e.g., sets of SBGN bricks instances (Rougny *et al*. 2021)). A complete use case comparing all human pathways in Reactome (Ragueneau *et al*. 2026) is available in the Supplementary Material.

## 7 Styling and style sheets

Using *momapy*, the graphical style of layout elements may be extensively customised. *momapy* supports the modification of common presentation attributes such as stroke and fill colors or line width, but also the application of advanced graphical effects such as shadows. These custom styles may be applied directly to the individual layout element objects, or using user-defined CSS-like style sheets (see Fig. 1B and C). *momapy* also includes a set of predefined style sheets, that mimic the graphical style of several common map editors such as Newt (Balci *et al*. 2021), SBGN-ED (Czauderna *et al*. 2010), or CellDesigner™ (Funahashi *et al*. 2008).

## 8 Rendering of maps

*momapy* may be used to render layouts of maps to images (e.g., SVG, PNG, JPEG, PDF files) or directly to the screen (e.g., OpenGL windows). The rendering is done offline using different alternative common backends (SVG-native, Skia (https://skia.org/), Cairo (https://www.cairographics.org/)), enabling the embedding of *momapy* in other applications on all platforms. Rendering with *momapy* supports all the aforementioned styles, including advanced graphical effects (depending on the backend).

## 9 Extending *momapy* with new formats and functionalities

*momapy* offers a set of abstract and concrete classes that may be easily extended for the fast development of new data models and glyphs. An example of such an extension is *momapy-bel* (available at https://github.com/adrienrougny/momapy_bel), which adds support for the Biological Expression Language (BEL) (Hoyt *et al*. 2018) to *momapy*. BEL is a textual language for the representation of relationships in biology, along their biological context. *momapy-bel* adds support for BEL to *momapy* by defining a new *BEL model* class that extends *momapy*’s base *model* class. Since *momapy* is built as a library, it can be freely used by third-party tools to work with molecular maps. Examples of such tools include *momapy-kb* (available at https://github.com/adrienrougny/momapy_kb), which allows users to integrate molecular maps into various types of knowledge bases (KB) including the Neo4j graph database (www.neo4j.com) and ASP programs (Gebser *et al*. 2019) so they can be easily queried or reasoned upon, and *momapy-draw* (available at https://github.com/adrienrougny/momapy_draw), a tool based on *momapy*’s rendering capabilities to draw SBGN and CellDesigner™ maps programmatically.

## 10 Software implementation and availability

*momapy* is implemented in Python (RRID:SCR_008394). Its code is freely available from https://github.com/adrienrougny/momapy under a GPLv3 license and is archived on Zenodo (https://doi.org/10.5281/zenodo.19088611). Complete documentation and a user manual are available at https://adrienrougny.github.io/momapy.

## Supplementary Material

btag352_Supplementary_Data
