# Supplementary material for: momapy: a Python library to work with molecular maps
Source: Bioinformatics. 2026 Jun 3;42(6):btag352. doi: 10.1093/bioinformatics/btag352 (PMC13282073; doi:10.1093/bioinformatics/btag352)
Supplement: btag352_Supplementary_Data [file btag352_supplementary_data.pdf]

momapy: a Python library to work with molecular maps
Supplementary material

Adrien Rougny Marek Ostaszewski Venkata Sagatopam
the COMMUTE consortium

May 8, 2026

1 Use case: comparison of the human pathways in Reactome with momapy

In this use case, we demonstrate how momapy can be used to compare a large set of maps. We find and visualise common modulations between human pathways in Reactome using *momapy*. We show that these operations can be performed easily with simple Python built-ins or *momapy* functions. This supplementary material is available as a runnable jupyter notebook at https://github.com/adrienrougny/momapy/blob/main/demos/reactome_analysis/main.ipynb.

[1]:
```
import pathlib
import shutil
import itertools
import tarfile
import urllib.request
import csv

import momapy.io.core
import momapy.sbgn.utils

import utils
```

1.1 Downloading the pathways

[2]:
```
input_dir_path = pathlib.Path("data/")
```

We remake the directory where we will download the pathways:

[3]:
```
if input_dir_path.exists():
    shutil.rmtree(input_dir_path)
input_dir_path.mkdir(parents=True)
```

We download the pathways, under the form of SBGN PD maps:

[4]:
```
sbgn_archive_url = "https://download.reactome.org/95/homo_sapiens.sbgn.tar.gz"
sbgn_archive_file_path = input_dir_path / "homo_sapiens.sbgn.tar.gz"
urllib.request.urlretrieve(sbgn_archive_url, sbgn_archive_file_path)
```

```python
with tarfile.open(sbgn_archive_file_path) as tar:
    tar.extractall(path=input_dir_path)
```

We download the list of pathways that maps pathways ids to pathways names:

[5]:
```python
list_of_pathways_file_path = input_dir_path / "list_of_pathways.csv"
```

[6]:
```python
list_of_pathways_url = "https://download.reactome.org/95/ReactomePathways.txt"
_ = urllib.request.urlretrieve(list_of_pathways_url, list_of_pathways_file_path)
```

[7]:
```python
pathway_id_to_name = {}

with list_of_pathways_file_path.open() as f:
    reader = csv.reader(f, delimiter="\t")
    for row in reader:
        pathway_id_to_name[row[0]] = row[1]
```

1.2 Comparing the pathways with momapy

We read all pathways. We want to compare their models, so we only extract those:

[8]:
```python
pathway_id_to_model = {}

for file_path in input_dir_path.glob("*.sbgn"):
    model = momapy.io.core.read(file_path, return_type="model").obj
    pathway_id_to_model[file_path.stem] = model

print(f"Got {len(pathway_id_to_model)} models")
```

```
Got 1358 models
```

We may compare different attributes of pathways (e.g., their entities, processes, modulations). Here we decide to compare their modulations, as they are the most complex objects. The identity of a modulation is defined by its type (e.g., stimulation), its source (entity pool or logical operator) and its target (stoichiometric process or phenotype). For each couple of different pathways, we compute the Jaccard index between their sets of modulations. In momapy, all model elements are frozen dataclass instances that can be hashed based on their identity, and stored in Python sets. Hence model elements, and sets of model elements, can be easily compared using built-in Python functions (set union and intersection, ==, etc.):

[9]:
```python
ATTRIBUTE = "modulations"

results = {}
for (pathway_id1, model1), (pathway_id2, model2) in itertools.combinations(
    pathway_id_to_model.items(), 2
):
    set1 = getattr(model1, ATTRIBUTE)
    set2 = getattr(model2, ATTRIBUTE)
    intersection = len(set1.intersection(set2))
    union = len(set1.union(set2))
```

```
    index = intersection / union if union > 0 else 0
    results[(pathway_id1, pathway_id2,)] = {"intersection": intersection, "index":␣
↪index}

print(f"Got {len(results)} indexes")
```

```
Got 921403 indexes
```

We sort the results by Jaccard index, and keep only the couples of pathways that have a minimum number of common modulations:

[10]:
```
MIN_INTERSECTION = 5

results = sorted(
    [item for item in results.items() if item[1]["intersection"] >= MIN_INTERSECTION],
    key=lambda item: item[1]["index"],
    reverse=True,
)

print(f"Got {len(results)} couples")
```

```
Got 41 couples
```

We print the 10 best results under the form "pathway1 | pathway2 | cardinality of intersection | Jaccard index":

[11]:
```
for (pathway_id1, pathway_id2), scores in results[:10]:
    print("*", pathway_id_to_name[pathway_id1], "|", pathway_id_to_name[pathway_id2],␣
↪"|", scores["intersection"], "|", round(scores["index"], 2))
```

```
* Signaling by NOTCH1 PEST Domain Mutants in Cancer | Signaling by NOTCH1
HD+PEST Domain Mutants in Cancer | 6 | 0.6
* Expression of BMAL (ARNTL), CLOCK, and NPAS2 | Heme signaling | 13 | 0.57
* DAG and IP3 signaling | Opioid Signalling | 20 | 0.44
* MyD88:MAL(TIRAP) cascade initiated on plasma membrane | MyD88 cascade
initiated on plasma membrane | 6 | 0.3
* Signaling by NTRK1 (TRKA) | MAPK targets/ Nuclear events mediated by MAP
kinases | 12 | 0.29
* Formation of the posterior neural plate | Formation of the anterior neural
plate | 5 | 0.26
* DAG and IP3 signaling | Anti-inflammatory response favouring Leishmania
parasite infection | 9 | 0.21
* Neddylation | KEAP1-NFE2L2 pathway | 12 | 0.2
* DAG and IP3 signaling | Activation of NMDA receptors and postsynaptic events |
9 | 0.19
* Formation of the nephric duct | Formation of intermediate mesoderm | 7 | 0.19
```

1.3 Visualising the results with momapy

The second result seems to show that heme signalling is somehow related to the circadian clock, which is an interesting observation. We could print the common modulations, but since we are working with maps, we can visualise them instead. To do this, we read the pathways, this time extracting the full maps, and display the layout elements representing the common modulations.

[12]:
```
pathway_id1, pathway_id2 = results[1][0]
print(f"Pathway 1: {pathway_id1} | {pathway_id_to_name[pathway_id1]}")
print(f"Pathway 2: {pathway_id2} | {pathway_id_to_name[pathway_id2]}")
```

```
Pathway 1: R-HSA-9931509 | Expression of BMAL (ARNTL), CLOCK, and NPAS2
Pathway 2: R-HSA-9707616 | Heme signaling
```

We extract the full maps, to have access to their layouts and layout model mappings:

[13]:
```
pathway1 = momapy.io.core.read(input_dir_path / f"{pathway_id1}.sbgn").obj
pathway2 = momapy.io.core.read(input_dir_path / f"{pathway_id2}.sbgn").obj
```

Reactome's SBGN output can be a bit messy so we first tidy the maps:

[14]:
```
pathway1 = momapy.sbgn.utils.tidy(pathway1)
pathway2 = momapy.sbgn.utils.tidy(pathway2)
```

We visualise the maps:

[15]:
```
utils.display(pathway1, scale=0.5)
```

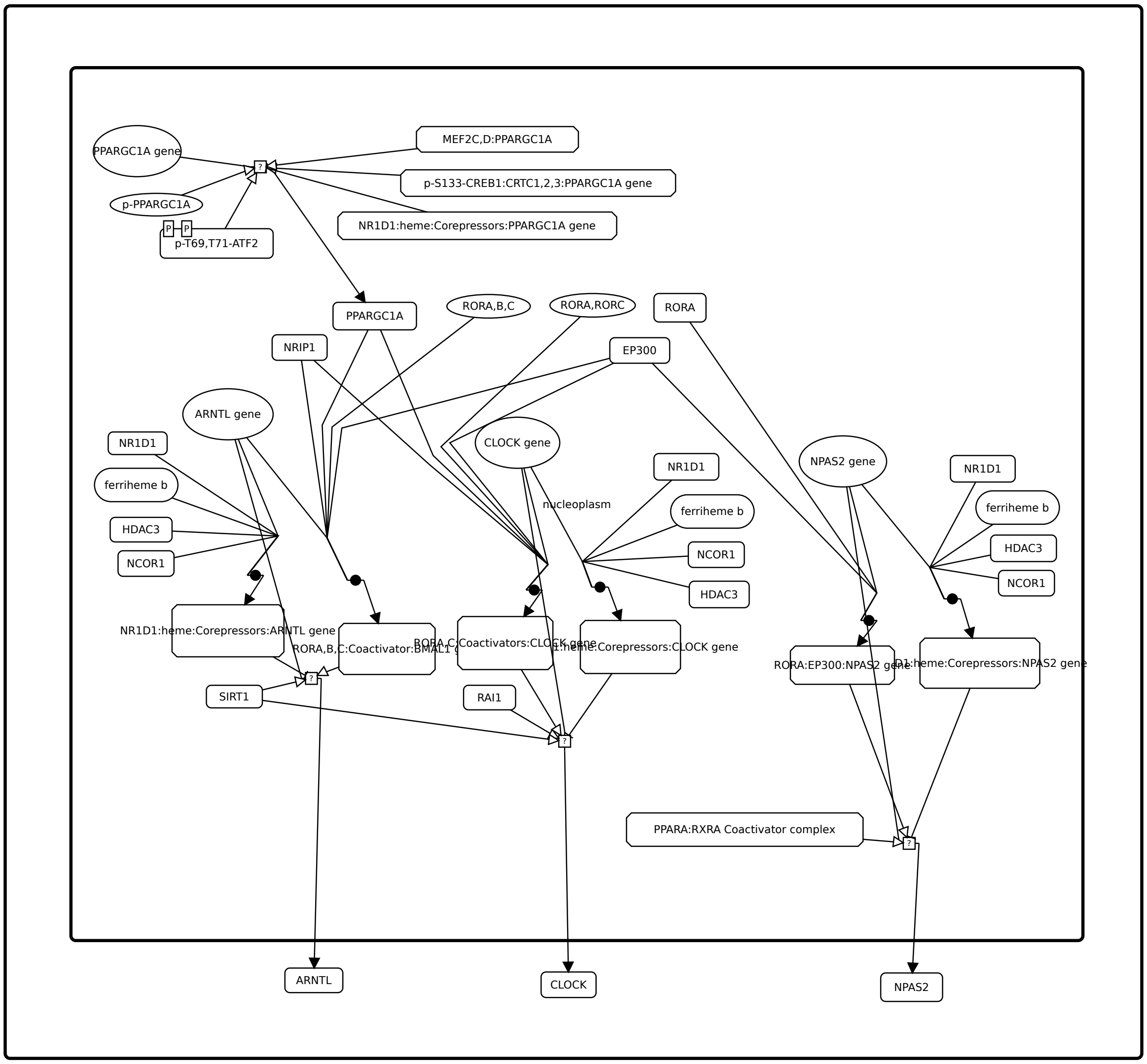


```
[16]: utils.display(pathway2, scale=0.5)
```

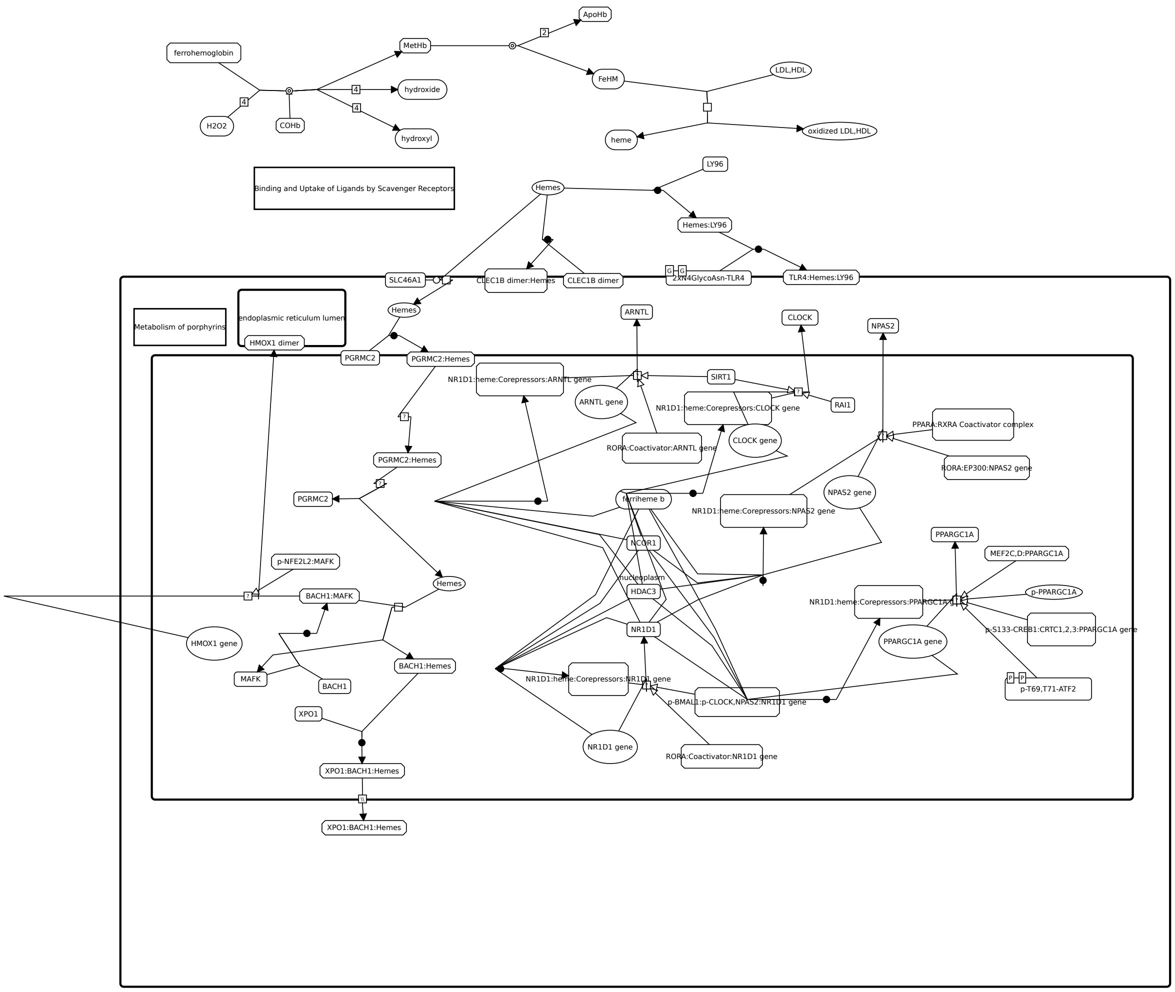

We get their common modulations once again:

[17]:
```
set1 = getattr(pathway1.model, ATTRIBUTE)
set2 = getattr(pathway2.model, ATTRIBUTE)
intersection = set1.intersection(set2)
```

Finally, we display the layout elements that represent the common modulations, that we take from the first pathway:

[18]:
```
for model_element in intersection:
    layout_elements = pathway1.layout_model_mapping.get_mapping(model_element)[0]
    utils.display(layout_elements)
```

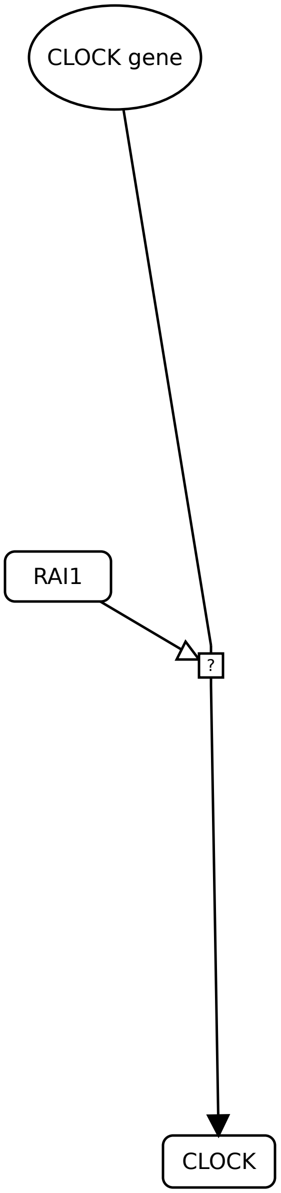
CLOCK gene
RAI1
?
CLOCK

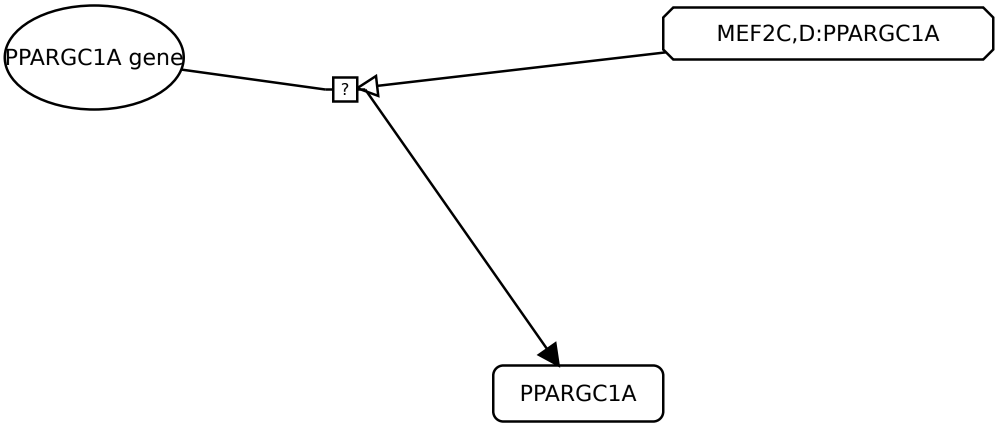
PPARGC1A gene
?
MEF2C,D:PPARGC1A
PPARGC1A

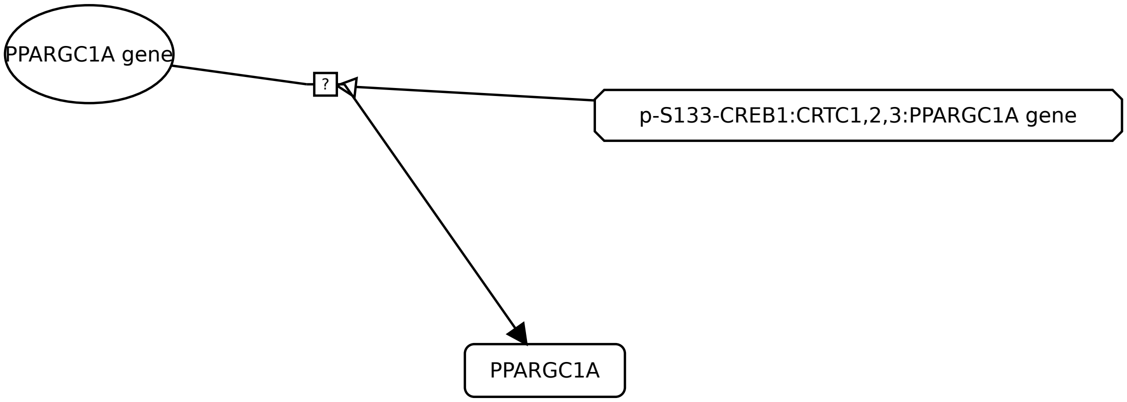
PPARGC1A gene
?
p-S133-CREB1:CRTC1,2,3:PPARGC1A gene
PPARGC1A

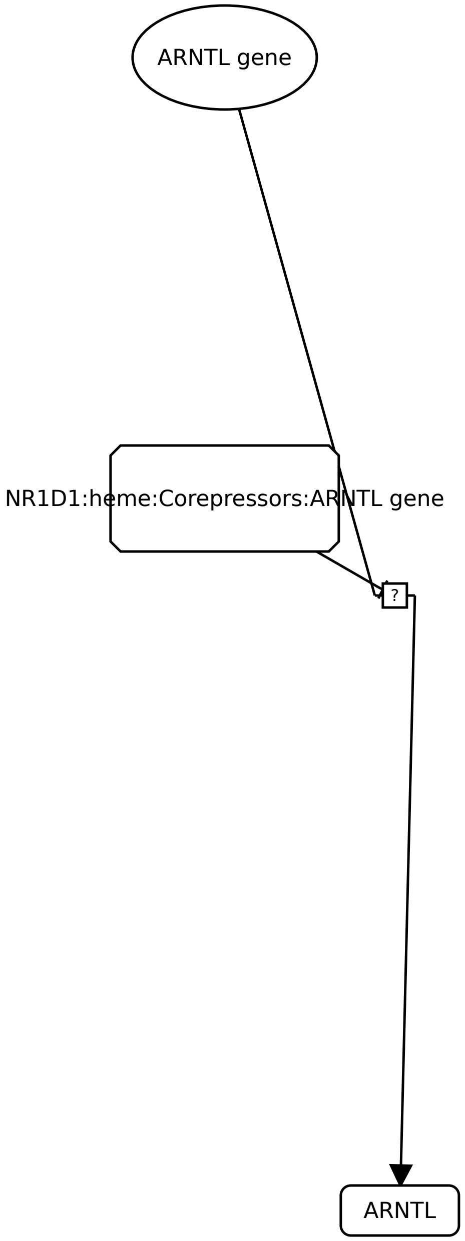
ARNTL gene
NR1D1:heme:Corepressors:ARNTL gene
?
ARNTL

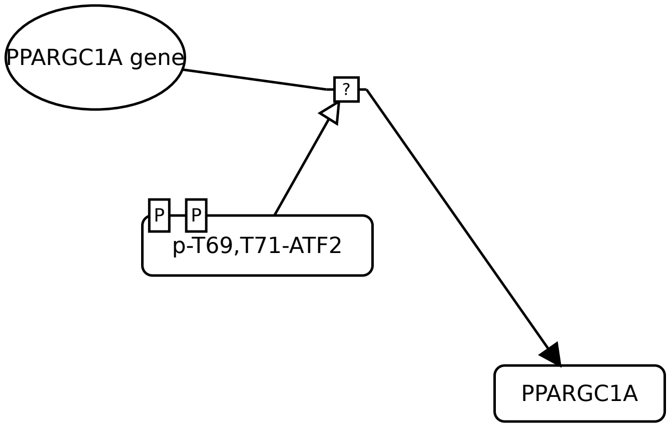
PPARGC1A gene
?
P
P
p-T69,T71-ATF2
PPARGC1A

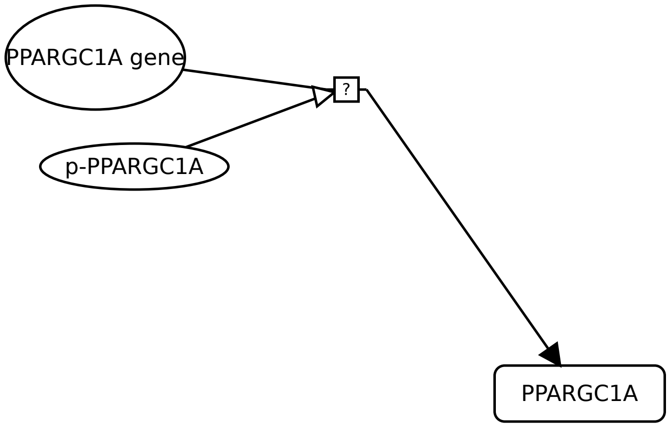
PPARGC1A gene
?
p-PPARGC1A
PPARGC1A

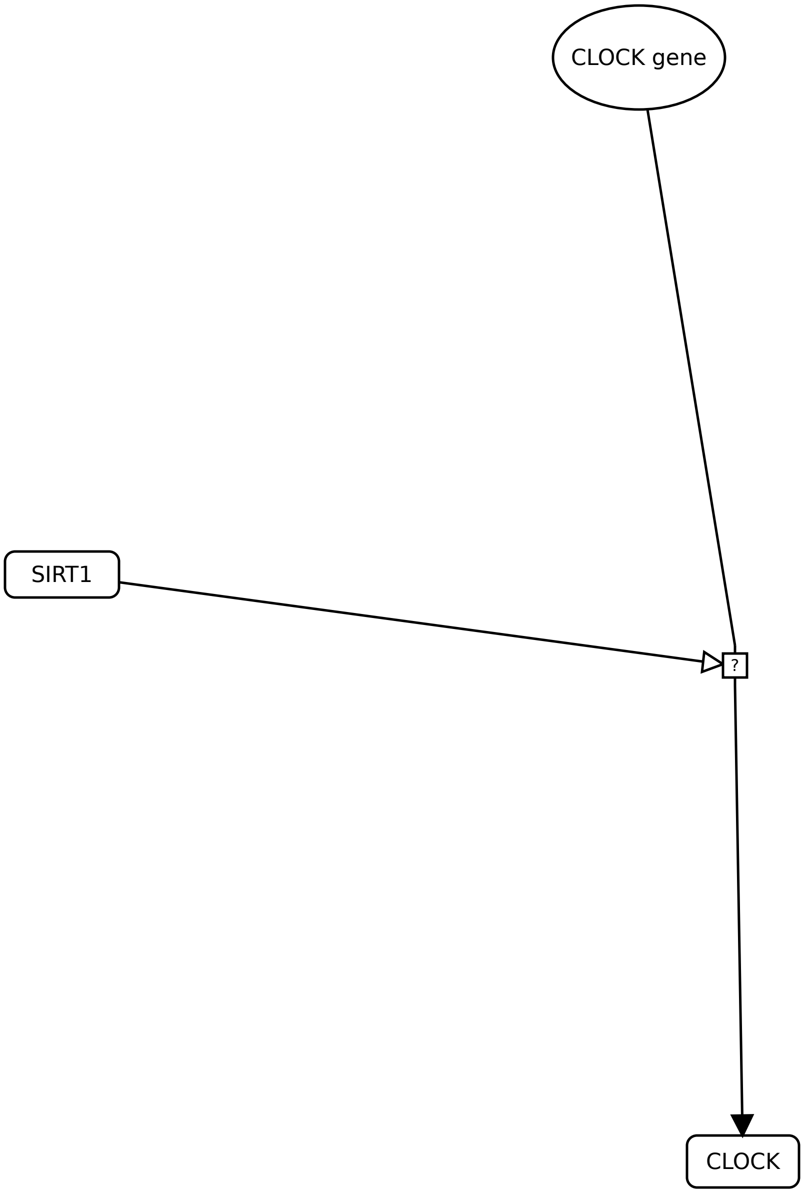
CLOCK gene
SIRT1
?
CLOCK

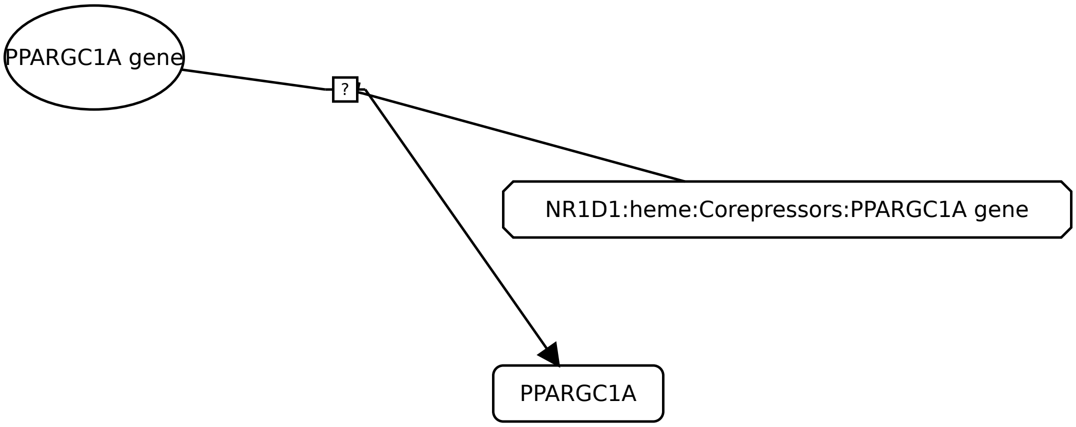
PPARGC1A gene
?
NR1D1:heme:Corepressors:PPARGC1A gene
PPARGC1A

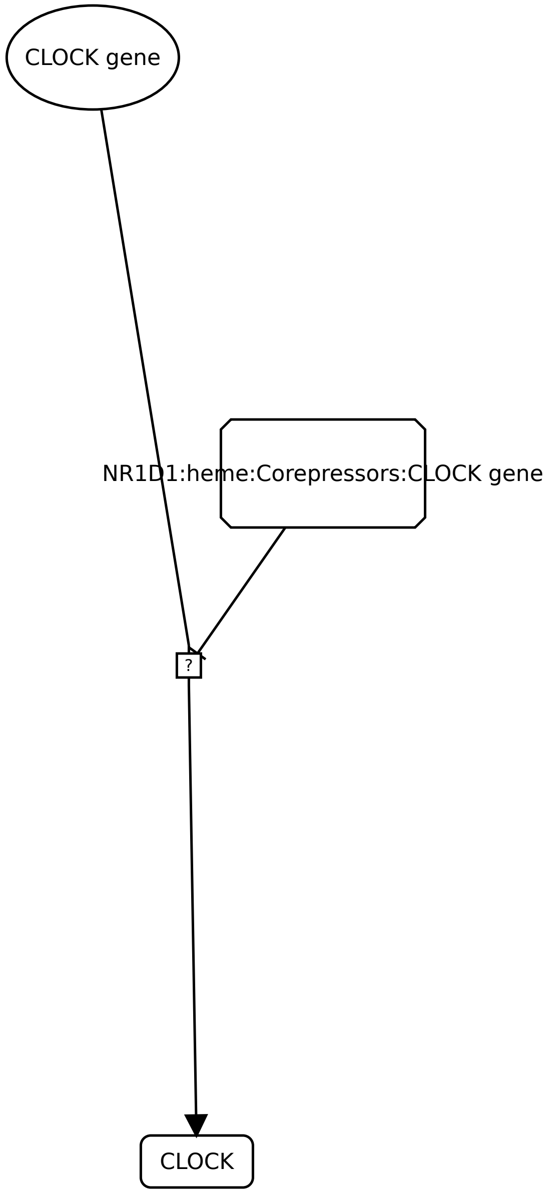
CLOCK gene
NR1D1:heme:Corepressors:CLOCK gene
?
CLOCK

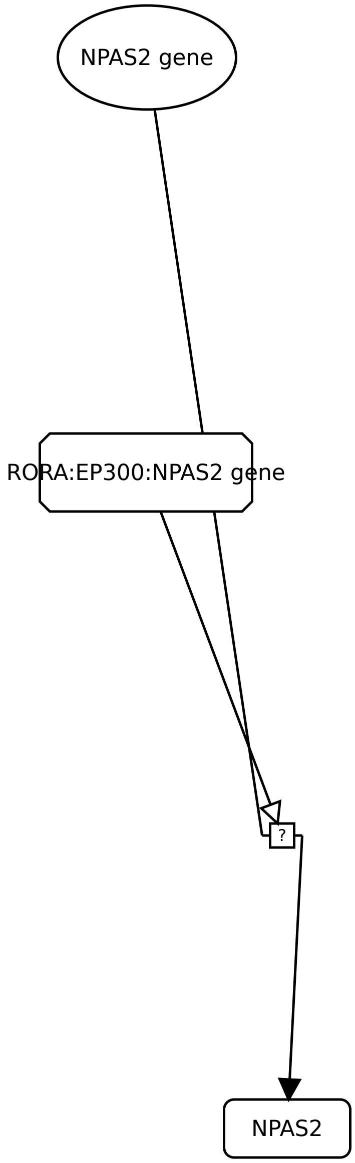
NPAS2 gene
RORA:EP300:NPAS2 gene
?
NPAS2

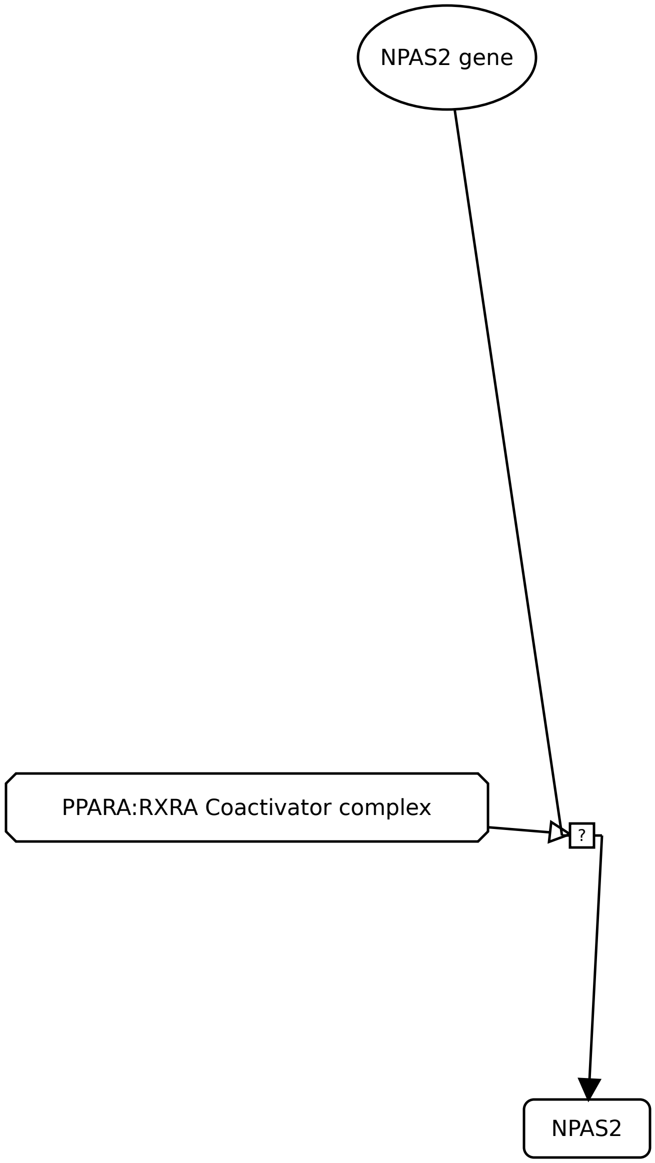
NPAS2 gene
PPARA:RXRA Coactivator complex
?
NPAS2

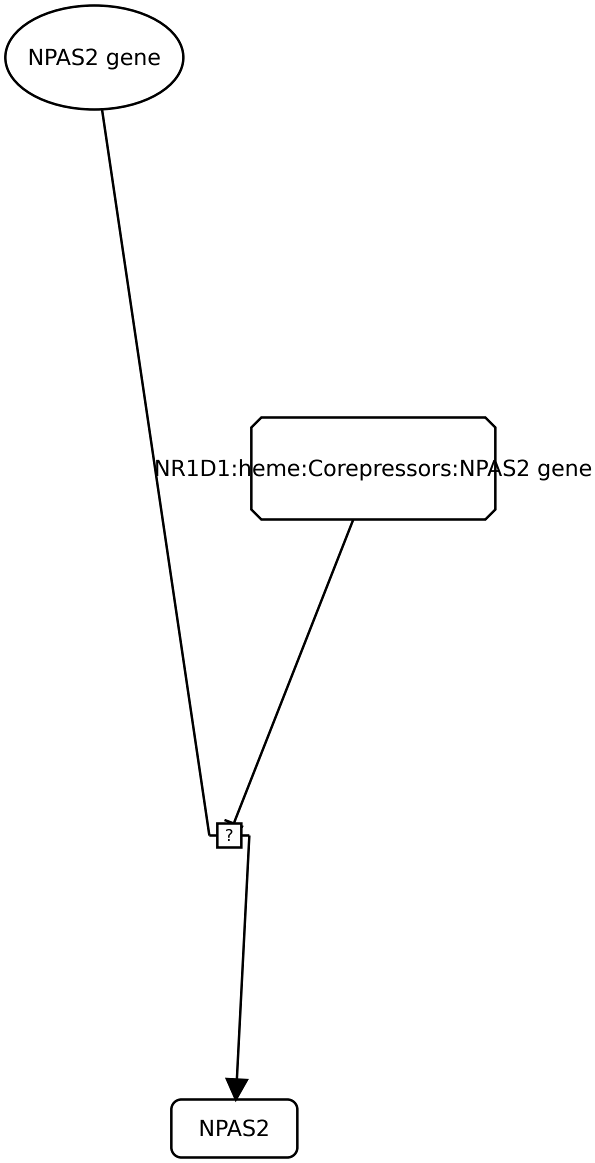
NPAS2 gene
NR1D1:heme:Corepressors:NPAS2 gene
?
NPAS2

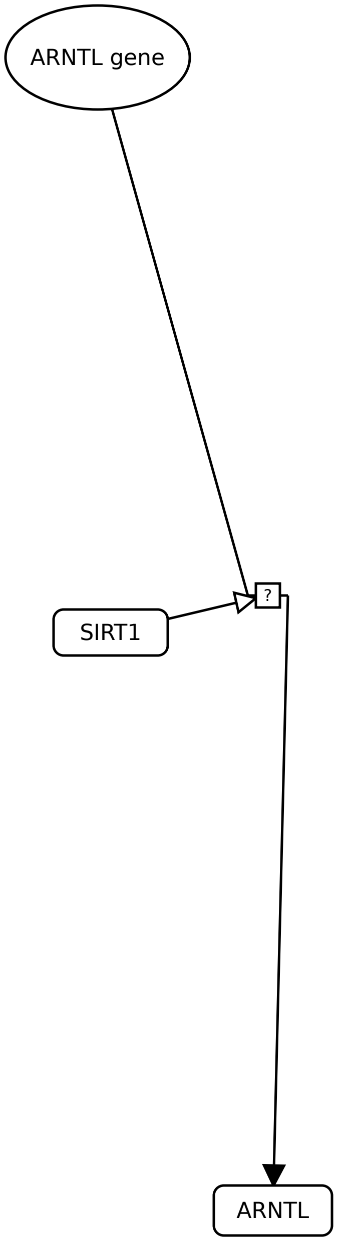
ARNTL gene
SIRT1
?
ARNTL
